# Continuous positive pressure ventilation combined with pulmonary surfactant in the treatment of neonatal respiratory distress syndrome

**DOI:** 10.12669/pjms.36.4.1963

**Published:** 2020

**Authors:** Jing Miao, Haitao Xie, Yanping Zhang, Xiaohui Guo, Min Cui

**Affiliations:** 1 Jing Miao, Department of Pediatrics, Binzhou People’s Hospital, Shandong, 256610, China; 2 Haitao Xie, Department of Pediatrics, Binzhou People’s Hospital, Shandong, 256610, China; 3 Yanping Zhang, Department of Pediatrics, Binzhou People’s Hospital, Shandong, 256610, China; 4 Xiaohui Guo, Department of Pediatrics, Binzhou People’s Hospital, Shandong, 256610, China; 5 Min Cui, Department of Pediatrics, Binzhou People’s Hospital, Shandong, 256610, China

**Keywords:** Continuous positive airway pressure treatment, Neonatal respiratory distress syndrome, Pulmonary surfactant, Therapeutic effect

## Abstract

**Objective:**

To analyze the clinical effect of nasal continuous positive airway pressure (CPAP) combined with pulmonary surfactant in the treatment of neonatal respiratory distress syndrome (NRDS).

**Methods:**

Eighty-two NRDS patients who received treatment from August 2017 to June 2019 in our hospital were selected and divided into a control group and an observation group using random number table, 41 in each group. The control group was treated with CPAP, and the observation group was treated with pulmonary surfactant injection besides CPAP. The therapeutic effect, blood gas index, mechanical ventilation parameters and occurrence of complications were compared between the two groups.

**Results:**

The total response rate of the observation group was 90.24%, which was significantly higher than 70.73% of the control group, and the difference had statistical significance (P<0.05). After treatment, the improvement of blood gas indexes of the observation group was better than that of the control group. The hospitalization time and duration of oxygen treatment of the observation group were shorter than those of the control group, and the hospitalization cost was higher than the control group (P<0.05). The difference of incidence of complications between the two groups was statistically significant (P<0.05).

**Conclusion:**

Endotracheal injection of pulmonary surfactant combined with CPAP in the treatment of NRDS can enhance the efficacy, promote the recovery of blood gas index, and reduce the parameters of mechanical ventilation and the incidence of complications, which is conducive to improving the respiratory function of the newborn. The therapy is worth application in the treatment of NRDS patients.

## INTRODUCTION

Neonatal respiratory distress syndrome (NRDS) refers to a kind of clinical syndrome in which symptoms such as dyspnea and respiratory failure occur within 4-12 h after birth. Due to the lack of pulmonary surfactant, alveolar collapse and reduced lung compliance happen, resulting in serious hypoxemia and lung injury.^1^-^3^ The epidemiological survey shows that NRDS is mostly found in premature infants, and its incidence is related to the gestational age and weight of children. The smaller the gestational age, the higher the incidence; the smaller the weight and the higher the mortality.^4^ A local study has also pointed out that the hospital mortality rate of NRDS children was as high as 10%-20%.^5^ At present, NRDS is mainly treated by nasal continuous positive airway pressure (CPAP) ventilation on the basis of supportive treatment, in order to improve children’s hypoxia symptom.^6^ However, mechanical ventilation treatment, due to its high technical requirements, is easy to induce complications and affect the prognosis and rehabilitation.^7^ In recent years, it has been pointed out that combining pulmonary surfactant with CPAP can further improve the ventilation status of NRDS children and improve the clinical treatment effect.^8^ In this study, 82 NRDS patients who were admitted to our hospital from August 2017 to June 2019 were taken as the study subjects and treated by CPAP and pulmonary surfactant combined with CPAP respectively to analyze the treatment effect and safety of CPAP combined with pulmonary surfactant in the treatment of NRDS, with the intention of providing a reference for the clinical treatment of NRDS.

## METHODS

### General data

Eighty-two NRDS patients who were admitted to our hospital from August 2017 to June 2019, 82 were selected. The inclusive criteria included meeting the relevant diagnostic criteria of NRDS in Practical Neonatology,^9^ having not receiving glucocorticoid treatment before delivery, and being transferred to intensive care unit within 12 hours after birth. Exclusive criteria included preterm infants who were suspected of pulmonary dysplasia, had congenital metabolic diseases, chromosomal abnormalities, congenital cardiopulmonary malformations, and severe anemia, had severe intravascular hemolysis shown by prenatal ultrasound, and had severe dyspnea at birth. The children were divided into a control group and an observation group, 41 in each group. In the control group, there were 21 males and 20 females, with a gestational age of 28-35 weeks (average (30.77±2.82) weeks) and a birth weight of 1630-2210 g (average (1941.72±43.46) g). In the observation group, there were 23 males and 18 females, with a gestational age of 29-36 weeks (average (31.13±3.01) years) and a birth weight of 1590-2250 g (average (1947.53±60.82) g). There was no significant difference between the two groups in general data (P>0.05). The study was approved by the medical ethics committee (Dated: 24 October 2019) of the hospital.

### Therapeutic method

The control group was treated by CPAP. The specialized nasal CPAP equipment was used. The concentration of oxygen was set as 40% ~ 60%. The gas flow parameter was set as 6 ~ 8 L/minutes. The pressure parameter was set as 3~8 cmH_2_O. The oxygen inhalation temperature was set as 36.8 ~ 37.3 °C. The vital signs of infants were closely monitored in the process of ventilation. The treatment such as conventional heat preservation, nutritional support and liquid supplement was performed as well. When the pressure of the CPAP equipment decreased, it was changed to mechanical ventilation after the blood oxygen concentration recovered normal. In addition to CPAP, the observation group was treated by pulmonary surfactant. The detailed treatment method is as follows. The breathing machine was removed. The airway secretion was cleaned. Poractant alfa injection (240 mg each one, Standard for registration of imported drugs: JX20030079) was injected at a dose of 100 mg/g, and it was heated to 37 °C. Then the infants received tracheal intubation at a supine position. After it was taken by a sterile needle, it was slowly injected at the bronchus bifurcation. Then the silicone tube was pulled out, and the rubber ball was pressed for two or three minutes. The drug was given once every twelve hours.

During the treatment, evidence-based nursing was carried out in the two groups. The specific operations were as follows.^10^ The first nursing care was to strengthen the observation of the disease condition, pay more attention to the risk factors of various diseases, and enhance the inspection of changes of vital signs and oxygenation index of the children. Secondly, reasonable mechanical ventilation treatment was performed according to the doctor’s advice, the lung protection strategy was strictly followed to keep the children’s respiratory tract unobstructed, and the airway management was strengthened. Thirdly, the preventive nursing interventions of complications were strengthened; after the operation of sputum suction and ventilation, combined with drug sensitivity test, sensitive antibiotics were given; the respiratory tract of the children was closely observed.

### Observation index

1. Before and 12 hours after treatment, GEMPremier 3000 automatic blood gas analyzer was used to detect the blood gas indexes of the two groups.
2. The clinical treatment effect was compared between the two groups.
3. The treatment indexes were compared between the two groups. The hospitalization time, duration of oxygen treatment and hospitalization time of the two groups were recorded by the charge nurse and analyzed and compared.
4. The occurrence of complications of the two groups was recorded.

### Efficacy criteria

The treatment was evaluated as markedly effective if the clinical symptoms of the child improved significantly, the blood gas analysis results showed normal, NCPAP parameters were down regulated, and the machine was ready to be removed. The treatment was considered as effective if the clinical symptoms improved, the blood gas analysis results were partially normal after treatment, but the machine was not removed for further observation. The treatment was evaluated as ineffective if the clinical symptoms did not improve or even aggravated. Total response rate = (number of markedly effective cases + number of effective cases)/total number of cases×100%.

### Statistical analysis

SPSS25.0 was used for data analysis. The counting data was expressed as [n%] and processed by X^2^ test. The measurement data was indicated by mean±SD and processed by t test. The value of P indicated that the difference had statistical significance.

## RESULTS

### Comparison of clinical effects between the two groups

The total response rate of the observation group was higher than that of the control group, and the difference was statistically significant (P<0.05, Table-I).

**Table-I:** Clinical efficacy between the two groups (%).

Group	Markedly effective	Effective	Ineffective	Total effective rate
Observation group	25(60.97)	12(29.27)	4(9.76)	37(90.24)
Control group	20(48.78)	9(21.95)	12(29.27)	29(70.73)
X^2^	/	/	/	5.641
P	/	/	/	0.027

### Comparison of arterial blood gas indexes between the two groups before and after treatment

No significant difference was observed in blood gas analysis between the two groups before treatment (P>0.05). After treatment, PaO_2_ and pH value of patients in the observation group were higher than those in the control group, and PaCO_2_ value was lower than that in the control group; the differences were statistically significant (P<0.05, Table-II).

**Table-II:** Blood gas analysis indexes before and after treatment between the two groups.

Group	Time point	PaO_2_(mmHg)	PaCO_2_(mmHg)	pH
Observation group	Before treatment	45.95±0.31	65.42±11.11	7.15±0.10
	After treatment	76.89±1.52^ab^	40.01±0.11^ab^	7.43±0.08^ab^
Control group	Before treatment	45.77±0.27	65.90±9.80	7.12± 0.09
	After treatment	70.64±1.61^a^	45.36±0.21^a^	7.32±0.07^a^

***Note:***

a P<0.05 compared with before treatment;

b P<0.05 compared with the control group 12 h after treatment.

### Comparison of treatment indexes between the two groups

The hospitalization time and duration of oxygen treatment of the observation group were shorter than those of the control group, and the hospitalization cost was higher than the control group (P<0.05, Table-III).

**Table-III:** Comparison of treatment indexes.

Group	Hospitalization time (d)	Duration of oxygen treatment (h)	Hospitalization cost (ten thousand yuan)
Observation group	13.68±1.57	54.68±11.22	2.63±0.21
Control group	16.38±2.50	98.76±13.22	1.32±0.16
t	2.369	1.682	3.627
P	0.021	0.035	0.016

### Comparison of incidence of adverse reactions between the two groups

In the observation group, there was one case of pulmonary air leak and one case of pulmonary hemorrhage; in the control group, there were five cases of pulmonary air leak, 4 cases of pulmonary hemorrhage and two cases of chronic lung diseases. The incidence of complications in the observation group was 4.88% (2/41), which was significantly lower than that in the control group (26.83% (11/41)). The difference was statistically significant (X^2^=6.373, P<0.05).

## DISCUSSION

At present, NRDS is mainly treated by hormone, positive pressure ventilation, surfactant and supportive therapy.^11^ The preventive application of glucocorticoid can promote the maturation of fetal alveoli and reduce the incidence of NRDS, but the long-term safety of the application of glucocorticoid is not clear. Although mechanical ventilation can improve the neonatal hypoxia symptoms, airway mucosa is easy to be damaged, and bacteria will propagate and migrate, which can cause ventilator-associated pneumonia. Therefore, CPAP is recommended to children with NRDS.^12^,^13^ CPAP can prevent children’s infection and enhance autonomous breathing ability to increase the residual air volume of lung function and the diameter of airway. However, if it is used alone, the recovery of blood gas index will be slow, which will increase the treatment time.^14^

In this study, pulmonary surfactant combined with CPAP was used. Pulmonary surfactant is mainly secreted by Type-II alveolar epithelial cells, which can effectively promote the reduction of surface tension of the lung, prevent further atrophy of the alveoli, inhibit lung lesions, and increase lung compliance.^15^,^16^ Moreover, pulmonary surfactant can effectively stabilize the intrapulmonary pressure, reduce the pressure of pulmonary capillaries, and prevent the liquid in capillaries from leaking into the lung. PS can improve the ventilation function of the lung, relieve the clinical symptoms of children, and alleviate the pain of children.^17^ Wang et al. found that the total effective rate was 94% by treating 50 NRDS children with pulmonary surfactant;^18^ after treatment, the pulmonary function of the children improved significantly, the parameters of ventilator decreased significantly, and the degree of alveolar inflation increased after treatment, suggesting that the clinical effect of pulmonary surfactant treatment was significant.

The results of this study showed that the total effective rate of the treatment in the observation group was higher than that in the control group, PaO_2_ and pH value were higher than those in the control group, and PaCO_2_ value was lower than that in the control group, suggesting that the combined treatment could increase the blood oxygen content of the newborn, improve the arterial blood gas, and significantly enhance the clinical efficacy; the results are consistent with the research conclusions of Siavashi.^19^ In addition, the research results also indicated that the incidence of complications in the observation group was significantly lower than that in the control group. The research report of Mc Pherson et al. suggested that pulmonary surfactant should be given as early as possible after birth as it could effectively reduce lung injury and reduce the incidence of complications, and it was consistent with the results of this study.^20^

The pathogenesis of NRDS is the absence of pulmonary surfactant. The application of pulmonary surfactant in the observation group fundamentally corrected the disease condition and improved the pulmonary ventilation function and pulmonary compliance. Moreover, surfactant reduced the surface tension of alveoli, which avoided the occurrence of alveoli collapse and thus improved the blood gas index of children.

## CONCLUSION

In conclusion, for patients with NRDS, the treatment of pulmonary surfactant combined with CPAP has a significant effect. It can enhance the efficacy, promote the recovery of blood gas index, and reduce the parameters of mechanical ventilation, so as to improve the respiratory function of the newborn. It is worth applying in the treatment process in the future.

### Authors’ Contribution

**JM & HTX:** Study design, data collection and analysis.

**JM, YPZ, XHG & MC:** Manuscript preparation, drafting and revising.

**JM:** Review and final approval of manuscript.

**JM:** Responsible and accountable for the accuracy or integrity of the work.
